# Influence of Physical Education Teachers on Motivation, Embarrassment and the Intention of Being Physically Active During Adolescence

**DOI:** 10.3390/ijerph16132295

**Published:** 2019-06-28

**Authors:** Rubén Trigueros, José M. Aguilar-Parra, Adolfo J. Cangas, Remedios López-Liria, Joaquín F. Álvarez

**Affiliations:** 1 Department of Psychology, Hum-878 Research Team, Health Research Centre, University of Almería, 04120 Almería, Spain; 2 Department of Psychology, Hum-760 Research Team, Health Research Centre, University of Almería, 04120 Almería, Spain; 3 Department of Nursing Science, Physiotherapy and Medicine, Hum-498 Research Team, Health Research Centre, University of Almería, 04120 Almería, Spain

**Keywords:** physical education, physical activity, motivation, embarrassment, adolescence

## Abstract

According to a WHO report (2018), more than 80% of adolescents do not do enough physical activity. Physical Education (PE) classes should be aimed at solving this problem. The present study aims to analyze the influence teachers have on motivation, embarrassment and intention to be physically active among their students. A total of 604 secondary school students participated in the study. Various statistical analyses were carried out to explain the causal relationships between the variables. The results revealed a positive relation between the autonomy support and the satisfaction of basic psychological needs (BPN), and a negative relation with the frustration of BPN. In contrast, perceived control revealed a positive relation with frustration of BPN, and a negative relation with the satisfaction of BPN. Satisfaction of BPN was negatively related to embarrassment and positively related to self-determined motivation. On the other hand, frustration of BPN was positively related to embarrassment and negatively with self-determined motivation. Embarrassment was negatively related to self-determined motivation, and the latter was positively related to intention to be physically active. Indeed, the study demonstrates the influence and the importance of PE teachers and of the motivational and emotional processes of adolescents during PE classes and the role they play in acquiring the habits of an active lifestyle.

## 1. Introduction

According to a WHO report [1], more than 80% of adolescents worldwide do not do enough physical activity, despite its numerous benefits to physical, mental and emotional well-being [2]. Physical Education (PE) classes should be aimed at solving this problem as the basic objectives of PE include teaching students to adopt regular physical activity habits in their free time. Thus, teachers play a key role given their influence, not only on the dynamics within PE classes, but also through the interaction they establish with students. Furthermore, teachers could directly impact student involvement in PE classes and whether students adopt active habits outside of the school setting and/or if those habits last throughout their lives [3].

Self-Determination Theory (SDT; [4]) suggests that social context can exert an influence on individuals through two very different interpersonal approaches: autonomy support and controlling style. Autonomy support involves fostering self-initiative and mental and physical self-development among students [5]. In contrast, controlling style emphasizes, for example, the use of external pressures, coercive methods and obligations, which are perceived by students as the origin of their behaviors, effectively undermining their self-initiative, effort and own self-knowledge [2]. Indeed, the role adopted by the teacher can significantly influence the development of students’ basic psychological needs (BPN) [6].

According to SDT, these BPN are defined as essential nutrients for personal development and well-being [2]. In this context, there are three psychological needs: autonomy, competence and relatedness [7]. However, a recent study conducted by González-Cutre et al. [8] proposed the incorporation of novelty as an additional BPN, which is defined as the innate tendency of the individual to search for new activities and experiences to achieve their complete development and well-being. In short, students who feel more autonomous when making decisions, that is, competent in their actions, supported and welcomed by the social group at hand, and who find different activities appealing, will experience a satisfaction of their BPN. Furthermore, such students tend to experience self-determined motivation, which is related to the learning of new habits, the commitment to learning, the improvement of interpersonal relationships and the manifestation of adaptive behaviors [4]. In contrast, if during PE classes students experience a feeling of abandonment, inadequacy in their actions, a lack of decision-making and a perception of excessively monotonous or repetitive activities, they will experience a frustration of their BPN. Such experiences tend to be marked by non-self-determined motivation, which is related to abandonment of an activity, a lack of both commitment and interpersonal relationships and, ultimately, the manifestation of maladaptive behaviors [6].

These BPN and the role of the teacher can have a significant influence on the motivation adopted by a student towards PE classes [4,5]. According to SDT, motivation can be self-determined or non-self-determined, where the former is related to behaviors based one’s own choices, decision-making capacity and personal initiative. In contrast, non-self-determined motivation is associated with participation in events due to acquired external pressures and/or obligations. This type of motivation leads to a lack of self-regulation of adaptive behavior as individuals tend to desist and distance themselves due to a lack of reward or external social recognition where there once had been. In contrast, self-determined motivation facilitates adaptation as it fosters self-regulation of behavior due to the fact that individuals tend to persist because of the satisfaction they obtain from doing the activity [7].

On the other hand, motivation is also affected by emotions, which constitute a powerful energizer that activates the inherent motivational processes within students [9]. In this context, various studies have analyzed the effect of different emotions on motivation [10]. Nonetheless, hardly any studies analyze the influence of the embarrassment that students experience during PE classes on motivation. In this regard, adolescence is a pivotal period linked to a number of physical, social and cognitive changes. These changes often generate certain feelings of rejection or shyness in different situations that take place during PE classes which impede or inhibit the students from carrying out a particular motor task [11]. One of the factors that favors student inhibition is adolescents’ own physical development, as they tend to compare themselves to other classmates. Moreover, the social relationships during adolescence is greater than during childhood, which increases the search for the feeling of belonging to a friendship group, causing them to flee from situations that may damage their social image [12]. In addition, the motivation of students toward PE classes can help to leading to increased leisure-time physical activity of youth, despite any bad experiences they might have [13]. In this sense, physical activity in leisure-time is often defined as a freely chosen activity involving physical efforts in one’s free time and that has health benefits for students [13]. From the salutogenic theory defines health as one’s location on the “ease/dis-ease” continuum with sense of coherence as a main construct in this model. According to Antonovsky [14], sense of coherence relates to the significance of “resistance resources” for prevention of health that based on an adequate self-evaluation, internal motivation for self-development through challenging leisure-time interests and social support when needed. In addition, Antonovsky formulated the existence of other term, general resistance resources refer to material resources (e.g., money, infrastructures) and personal (e.g., social support, intelligence, self-esteem). All of those should be considered important as well as opportunities to study, access to friends or educationally created opportunities for leisure-time activities [15].

At present, existing studies on the topic of PE classes have primarily focused on the positive aspect of SDT, analyzing how autonomy support from teachers positively influences satisfaction of BPN [16] and motivation towards PE classes [17], and, in turn, how the latter affects motivation to engage in physical activity and the intention to be physically active [18]. However, a new line of research has recently emerged which focuses on analyzing the negative consequences of the controlling teaching style on different variables. Thus, such studies are able to identify the negative effects this style has on the satisfaction of BPN [19], self-motivation [20] and learning itself [21]. Nevertheless, the studies cited have not addressed the role of the teacher in both types of teaching approaches (autonomy support and controlling style), and only a small number of other studies have actually done so (e.g., Jang et al. [22]). Therefore, the present study aims to analyze both approaches to elaborate a general perspective of the influence of PE teachers on both the BPN of students and participation and commitment to PE classes, while also seeking to contribute data on the Spanish context that is similar to those obtained by studies conducted in other countries (e.g., Jang et al. [22]). Similarly, we also find limitations with regard to the studies that comprise the measure of BPN. More specifically, such studies have analyzed the effects of satisfaction of BPN on the perception and motivation of students towards PE classes, using them as predictors of frustration. However, this method proves non-viable considering the items on the scale measuring satisfaction of PN only include positive psychological experiences, meaning it is highly unlikely that the negative aspects of these experiences are represented [23].

Regarding the aspect of embarrassment, to our knowledge no studies have analyzed the influence of this emotion on the motivation of students during PE classes. However, a series of approximations have been carried out which are related to the emotion of embarrassment in the context under study (e.g., Gil-Madrona and Martínez-López [24]). These studies highlight the emotions experienced by students during PE classes and the effects they have on students’ psychological well-being [25], prevention of high-risk behaviors [26], social-academic adjustment [27], competence [28] and happiness [25]. For this reason, it is essential to analyze the emotional aspects involved in an evaluation conducted by students themselves. The emotional processes mentioned begin with the perception of events and phenomena in reality that culminate in opinions, attitudes and beliefs that condition the social behavior of students. More specifically, embarrassment is an emotion that focuses on the self (e.g., some issue with body image of pubertal pupils) and is characterized as producing a negative effect that brings about a change in behavior, causing a detriment to the individual’s perception of their own adaptability in both social and personal contexts [29].

**Figure 1:**
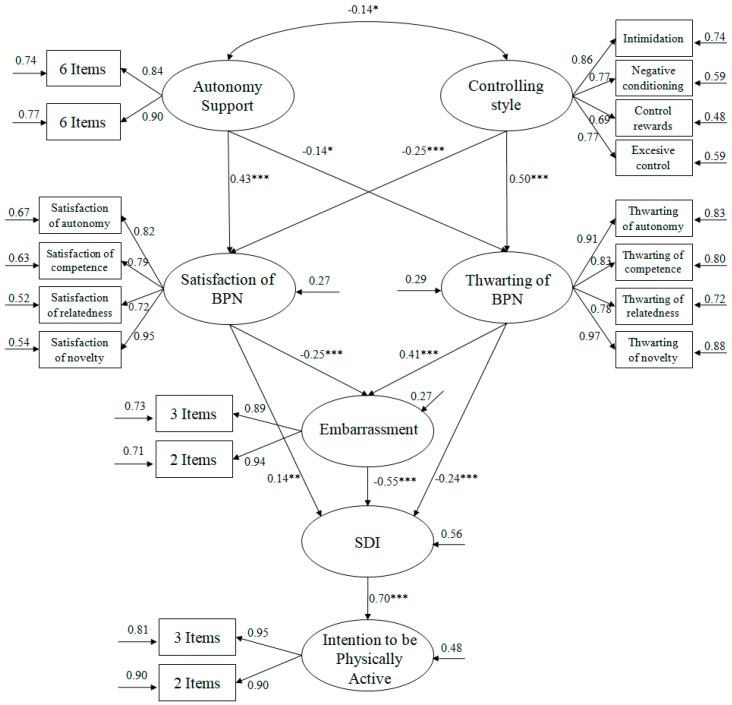
Of structural equations showing the relationships between the different variables. All parameters are standardized and statistically significant. The variances explained are shown above the small arrows. Note: *** *p* < 0.001; ** *p* < 0.01; * *p* < 0.05.

Taking these considerations into account, the present study was designed based on the following hypotheses (see Figure 1): (1) The autonomy support provided by the teacher will positively predict satisfaction of BPN and, at the same time, will negatively predict thwarting of BPN; (2) The controlling style of the teacher will negatively predict satisfaction of BPN and will positively predict frustration of BPN; (3) Satisfaction of BPN will positively predict self-determined motivation and negatively predict embarrassment; (4) Thwarting of BPN will negatively predict self-determined motivation and positively predict embarrassment; (5) Embarrassment will negatively predict self-determined motivation; (6) Self-determined motivation will positively predict the intention to be physically active.

## 2. Method

### 2.1. Participants

The study included the participation of 604 secondary school students, 321 boys and 283 girls between the 13 and 19 years of age (Mean (M) = 15.73; Standard Deviation (SD) = 1.30), from various schools in a province of Spain. The students attended two PE classes, each with a duration of one hour. The classes were conducted respecting equality of both students’ rights and duties. The inclusion criterion for participation in the study was: to provide informed advice signed by parents or legal guardians. On the other hand, the students were awarded by their PE teacher with 0.25 points in the final grade of the subject, in order to encourage their participation in this study. The completion of the questionnaires was carried out by those students who attended class that day, representing the 85.6% of the secondary school students.

### 2.2. Instruments

Perceived autonomy support: The instrument utilized was the Spanish version of Perceived Autonomy Support Scale for Exercise Settings by Hagger et al. [30], validated by Moreno et al. [31] for PE in the Spanish context. This scale is comprised of 12 items that evaluate one single factor of autonomy support. The questionnaire is based on a Likert scale from 1 (totally disagree) to 7 (totally agree).

Controlling style. The tool utilized was the PE Spanish version by Trigueros et al. [3] of Controlling Coach Behaviors Scale (CCBS) by Bartholomew et al. [32]. This questionnaire included 15 items divided among four factors which measure control via rewards, negative conditioning, intimidation and excessive personal control. The questionnaire is based on a Likert scale from 1 (totally disagree) to 7 (totally agree).

Satisfaction of basic psychological needs: The instrument used was the Spanish version of Basic Psychological Needs in Physical Education [33], validated and adapted for the PE context in Spain by Menéndez and Fernández-Río [34]. Furthermore, the study of Trigueros et al. [23] incorporated the items relating to the novelty developed by González-Cutre et al. [8] into the Menéndez and Férnandez-Rio instrument. The scale is comprised of 18 items divided among satisfaction of autonomy, competence, relatedness and novelty. In this case, the questionnaire is based on a Likert scale from 1 (totally disagree) to 7 (totally agree).

Frustration of BPN: The tool utilized was the Thwarting of BPN in PE Scale by Trigueros et al. [35]. The scale is comprised of 17 items divided among thwarting of autonomy, competence, relatedness and novelty. The questionnaire is based on a Likert scale from 1 (totally disagree) to 7 (totally agree).

Embarrassment: In order to measure embarrassment, we utilized the factor of the same name taken from the Emotions in PE Questionnaire (Cuestionario de Emociones en Educación Física (CEEF) in Spanish) by Trigueros et al. [36]. The questionnaire is presented under the heading “During PE classes…” and includes 34 items divided among eight factors, four of which with positive valence and four with negative valence. More specifically, the embarrassment factor is comprised of five items (e.g., I feel embarrassed when I do exercise in class.) The questionnaire is based on a Likert scale from 1 (totally disagree) to 7 (totally agree).

The fit indices displayed by the scale were adequate (Trigueros et al. [36]): *χ*^2^/*gl* = 2.04, *p* = 0.001; Comparative Fit Index (CFI) = 0.94; Tucker Lewis Index (TLI) = 0.94; Root Mean Square Error of Approximation (RMSEA) = 0.048; Standardized Root Mean Square Residual (SRMR) = 0.048. The correlation between the factors ranged between −0.35 and 0.84, all of which were statistically significant. The correlation was positive between factors with the same valence, and negative between those with a different valence. The Cronbach’s alpha values for the 8 factors were greater than 0.70, among which embarrassment was 0.84.

Motivation: The version utilized was the Spanish version of Perceived Locus of Causality Revised (PLOC-R) by Vlachopoulos et al. [33], validated and adapted to the PE context of Spain by Trigueros et al. [37]. The scale includes 23 items divided among six factors which measure intrinsic motivation, integrated regulation, identified regulation, introjected regulation, external regulation and demotivation. The questionnaire is based on a Likert scale from 1 (totally disagree) to 7 (totally agree).

The self-determination index was utilized (SDI; [38]). It was calculated based on the following formula: 3 × intrinsic motivation, 2 × integrated regulation, 1 × identified regulation, −1 × introjected regulation, −2 × external regulation and −3 × demotivation. This index has been proven to be valid and reliable in various works in which it was used to obtain a value that could quantify levels of self-determination.

Intention to be physically active: The version utilized was the Spanish version of Intention to be physically active by Hein et al. [19], validated and adapted to the PE context in Spain by Moreno et al. [39]. This scale is comprised of five items that measure said intention. The questionnaire is based on a Likert scale from 1 (totally disagree) to 7 (totally agree).

### 2.3. Procedure

Firstly, as the students were under age, a written authorization was requested from both the school and the parents of the participants. Beforehand, teachers were informed that the collection of data would last two weeks and that the questionnaires would be administered before the beginning of PE classes. Subsequently, students were told that they would be participating in a research investigation on motivation towards PE classes and that data collection would take place before the beginning of classes. The questionnaires were answered anonymously. This study was carried out in accordance with the recommendations of the American Psychology Association. The entire experiment was conducted in accordance with the Declaration of Helsinki. Ethics approval was obtained from the Research Ethics Committee of the University of Almeria, Spain (Ref. UALBIO 2019/014).

### 2.4. Data Analysis

The present study carried out descriptive statistical analyses, bivariate correlations and reliability analyses using statistics program SPSS v24 (IBM, Armonk, NY, USA). In addition, a structural equations model was analyzed (SEM) using statistics program AMOS v19 (IBM, Armonk, NY, USA).

In order to analyze the hypothesized model (Figure 1) the method utilized was the maximum likelihood estimation, in conjunction with a bootstrapping procedure. The estimators were not affected and were therefore considered robust. With the objective of accepting or rejecting the tested model, a set of fit indices were taken into consideration: Hair, Black, Babin, Anderson and Tatham, [40]: *χ*^2^/*gl*, CFI (Comparative Fit Index), TLI (Tucker Lewis Index), RMSEA (Root Mean Square Error of Approximation) plus its confidence interval (CI) at 90%, and SRMR (Standardized Root Mean Square Residual). Given that *χ*^2^ is very sensitive to sample size, *χ*^2^/*gl* was utilized, where values below 3 were considered acceptable. The incremental indices (CFI-TLI) display good fit, with values equal to or greater than 0.95, while error indices (RMSEA and SRMR) are considered acceptable with values equal to or less than 0.06.

## 3. Results

### 3.1. Preliminary Analysis

The descriptive statistics, bivariate correlations and reliability analysis obtained using Cronbach’s alpha can be observed in Table 1. The highest average scores were obtained for autonomy support and for satisfaction of BPN. In contrast, the lowest average scores were obtained for embarrassment and frustration of BPN.

With regard to the correlation analyses, the results revealed a positive association between similar factors and a negative association between those that were different, which is in keeping with the postulates of SDT.

### 3.2. Structural Equations Model

Prior to testing the hypothesized model using an SEM and analyzing the relationships existing between the variables belonging to the model, the number of latent variables was reduced, whereby each one had at least two indicators due to the complexity of the model [41]. More specifically, the latent variables used were: the four indicators included in thwarting of BPN (thwarting of autonomy, novelty, competence and relatedness, Trigueros et al. [35]); the four indicators included in satisfaction of BPN (satisfaction of autonomy, novelty, competence and relatedness González-Cutre et al. [8]); and the four indicators included in teacher control (intimidation, excessive personal control, use of rewards and negative conditioning, Trigueros et al. [3]). In addition, in the case of autonomy support, it was necessary to divide the 12scale items into two indicators, which was also required for the five items of embarrassment and intention. The procedure described was necessary to identify the model, as suggested by McDonald and Ho [41].

The hypothesized model for the predictive relationships (Figure 1) shows that the fit indices were adequate: *χ*^2^ (80, *N* = 604) = 209.614, *χ*^2^/*gl*= 2.87, *p* < 0.001, IFI = 0.95, CFI = 0.95, RMSEA = 0.047. (IC 90% = 0.035–0.057), SRMR = 0.039. The results fit the established parameters, meaning the proposed model can be accepted as suitable. Similarly, the contribution of each one of the factors to the prediction of other variables was examined by means of standard regression weights.

In Figure 1, it can be observed how autonomy support positively predicted satisfaction of BPN and negatively predicted thwarting of BPN. In contrast, perceived control positively predicted thwarting of BPN, while satisfaction of BPN was negatively predicted. Satisfaction of BPN negatively predicted embarrassment and positively predicted self-determined motivation, while thwarting of BPN positively predicted embarrassment and negatively predicted self-determined motivation. Finally, embarrassment negatively predicted self-determined motivation, which, in turn, positively predicted intention to be physically active.

## 4. Discussion

The present study sought to analyze how a teacher’s interpersonal style can influence BPN, embarrassment, self-determined motivation and the intention to be physically active among secondary school students in PE classes. This work is the first to address the teacher’s role from the perspectives of autonomy support and controlling style and the influence of this role on thwarting and satisfaction of BPN, which are also studied in relation to the subject of PE classes for the first time. The dynamic which exists between teachers and BPN proves of utmost importance given the impact teachers have on the emotional, social and psychological development of students [5]. Also proving of equal importance is the vital role of students’ BPN, as they constitute basic and universal requirements common to all human beings, which can have a series of positive and negative consequences at psychological, behavioral and emotional levels, depending on whether they are satisfied or not [10].

Various studies on the subject of PE have confirmed the positive effect of autonomy support in relation to satisfaction of BPN of PE students, and, in turn, the effect of the latter on motivation towards said classes [4,5]. However, hardly any research has taken into account the negative aspects also present during PE classes, such as controlling teaching style and thwarting of BPN. Information on these two aspects proves crucial because, depending on the teacher’s role and students’ perception of their PE classes, they can exert a negative influence on the acquisition of future behaviors. In this line, researchers have observed that a low score for the satisfaction of BPN constitutes a predictor of thwarting. Nevertheless, this statement proves insufficient since the items on the BPN satisfaction and autonomy support scales merely include positive experiences, making it highly unlikely that any negative aspects of these experiences are represented [16].

As for the results, it can be observed that autonomy support positively predicted satisfaction of BPN and negatively predicted thwarting of BPN. In contrast, perceived control positively predicted thwarting of BPN and negatively predicted satisfaction of BPN. These findings can be compared with similar studies conducted in both Spain and other countries in which it has been shown that autonomy support is positively related to satisfaction of BPN [42] and negatively to thwarting of BPN [43]. In terms of previous studies on the subject of PE which link controlling style to satisfaction and thwarting of BPN, hardly any information can be found. The only available data that is somewhat related come from the fields of physical activity and sports, in which various works conducted by Cantú-Berrueto et al. [44] and Gurrola et al. [45] demonstrate that a controlling teaching style is positively related to thwarting of BPN and negatively to satisfaction of BPN. However, the fact remains that these specific studies display limitations, prompting the present study to take certain aspects into account, which resulted in a model in which both types of teacher interpersonal relationships are linked to the satisfaction and thwarting of BPN. Consequently, the findings of the present study are in line with those presented in previous studies and are also in keeping with the postulates of SDT. The results for the shared role between teachers and BPN established in this study can be explained by the fact that if students perceive a sense of both freedom to perform and decision-making power, their perceived competence, psychological well-being and satisfaction of BPN will all benefit. However, if a teacher’s behavior is autocratic or restrictive or places pressure on students, the group will feel oppressed, incapable and rejected because their BPN will tend to be frustrated.

The results also revealed that frustration of BPN negatively predicted self-determined motivation and positively predicted embarrassment. In contrast, satisfaction of BPN negatively predicted embarrassment and positively predicted self-determined motivation. These results are similar to those of previous studies related to motivation [43]. It was determined that feeling competent and capable during class activities and in control of one’s own actions and enjoying good interpersonal relationships established between members of a team and/or the teacher all help students feel self-determined motivation towards PE classes [17]. In regards to embarrassment, the results are in accordance with the postulates established in SDT [4] and by various studies that have analyzed the effects of BPN on emotional and psychological well-being [23,46]. These findings can be interpreted with the aid of the idea presented by Deci and Ryan [4] which states that the thwarting of BPN can trigger a series of maladaptive consequences (e.g., embarrassment) that can lead to inhibition and/or detrimental behaviors to personal well-being.

The results of the study also revealed that self-determined motivation was negatively predicted by embarrassment and that it positively predicted the practice of physical activity during free time. These findings are in keeping with the postulates established by SDT, in which Hagger and Chatzisarantis [47] established that motivation towards PE classes positively predicted the practice of physical activity. Both contexts are closely related considering that among the goals of PE classes are the acquisition of active habits outside of the school setting [48,49]. Therefore, it is fundamental that PE classes be motivating and appealing in order that students may extrapolate the positive experiences during class time to other physical activity contexts. Thus, teachers should not focus exclusively on the student’s technical, strategic, and tactical skills, but should also focus on developing effective relationships with their students, creating a climate conducive to experiencing positive emotions during physical education classes. Initially, the teachers may feel the need to be somewhat controlling and apply pressure to students so that they will put effort into the motor task, but they need to realize that this can often damage their perception to de physical education classes and thus physical activity. Instead, striving to instruct students in autonomy-supportive ways, such as acknowledging their negative feelings toward certain tasks, providing them with specific rationales about why the basic skills are helpful for their lives, and providing some choice when possible, will yield better results. Finally, the autonomy support of teachers to develop effective relationships with their students, which could have an impact on resulting in improved performance and goals.

Finally, embarrassment negatively predicted self-determined motivation. This result is in consonance with a recent study carried out by Leisterer and Jekauc [50] in the context of PE. This qualitative study showed how the embarrassment is very present among PE students when they are doing different exercises and motor tasks. Furthermore, this result can be to studies conducted in a sports context in which those athletes that felt self-conscious about their physique ceased participating in training or competition [51] because their motivation and perception of their activity had been negatively affected. In short, whether it be because of their physique, lack of capacity or fear of being judged, the feeling of embarrassment can negatively affect students’ motivation during classes, leading them to avoid the activity.

Indeed, the results obtained in the present study support the postulates of SDT by introducing new variables and demonstrating their applicability in Spanish society. The model appears to display good robustness and a capacity for generalization to other societies and ages. Moreover, it helps us to better understand the role played by teachers in the consolidation of habits of a healthy lifestyle. Nevertheless, the fact that this is a correlational study does not allow us to extrapolate cause-effect relationships and the results obtained could be interpreted in multiple ways, depending on the perspective of the individual. Thus, an attempt was made to present different possibilities for the purpose of explaining the existing relationships between the variables. In this line, future studies should thoroughly analyze the results obtained by using intervention studies that better clarify the relationship between the different study variables. Furthermore, it would also be useful to determine the influence of motivation and embarrassment given the variability of how they are perceived as adolescents grow up and progressively make their own decisions independently.

## Figures and Tables

**Table 1:** Statistics and correlations between all variables.

Factors	*M*	DT	Range	α	1	2	3	4	5	6	7
1. Controlling Style	2.16	0.86	1–7	0.83		−0.10 *	0.48 **	−0.27 **	0.37 **	−0.44 **	−0.18 **
2. Autonomy Support	4.82	1.25	1–7	0.87			−0.22 **	0.39 **	−0.29 **	0.31 **	0.35 **
3. Thwarting of BPN	1.99	1.04	1–7	0.89				−0.42 **	−0.46 **	−0.56 **	−0.18 **
4. Satisfaction of BPN	4.85	1.10	1–7	0.85					−0.39 **	0.48 **	0.59 **
5. Embarrassment	1.62	0.86	1–7	0.91						−0.67 **	−0.41 **
6. SDI	13.46	13.72	1–7								0.67 **
7. IPA	3.50	1.25	1–5	0.92							

Note: SDI = Self-Determination Index; BPN = Basic Psychological Needs; IPA = Intention to be Physically Active; ** *p* < 0.01; * *p* < 0.05.
